# Sex-specific factors associated with acceptance of smartwatches among urban older adults: the Itabashi longitudinal study on aging

**DOI:** 10.3389/fpubh.2024.1261275

**Published:** 2024-02-23

**Authors:** Naoki Deguchi, Yosuke Osuka, Narumi Kojima, Keiko Motokawa, Masanori Iwasaki, Hiroki Inagaki, Fumiko Miyamae, Tsuyoshi Okamura, Hirohiko Hirano, Shuichi Awata, Hiroyuki Sasai

**Affiliations:** ^1^Research Team for Promoting Independence and Mental Health, Tokyo Metropolitan Institute for Geriatrics and Gerontology, Tokyo, Japan; ^2^Department of Frailty Research, Center for Gerontology and Social Science, Research Institute, National Center for Geriatrics and Gerontology, Obu, Japan; ^3^Division of Preventive Dentistry, Department of Oral Health Science, Graduate School of Dental Medicine, Hokkaido University, Sapporo, Japan; ^4^Integrated Research Initiative for Living Well with Dementia, Tokyo Metropolitan Institute for Geriatrics and Gerontology, Tokyo, Japan

**Keywords:** wearable healthcare device, mobile health, smart wearables, health promotion, technology innovativeness

## Abstract

Smartwatches (SW) are wearable devices that support daily life and monitor an individual’s health and activity status. This information is utilized to promote behavior modification, which could help prevent chronic diseases and manage the health of older adults. Despite being interested in SWs, older adults tend to decrease their SW usage as they age. Therefore, understanding the acceptance of SWs among older individuals can facilitate individual health management through digital health technology. This study investigated the factors associated with the acceptance of SWs among older adults in Japan and the variations in the factors by sex. This study utilized data from the 2022 Itabashi Longitudinal Study on Aging, an ongoing cohort study conducted by the Tokyo Metropolitan Institute for Geriatrics and Gerontology. We included 899 eligible individuals aged ≥65 years. Participants were classified into three groups: possessing SW (possessor group), not possessing SW but interested in possession in the future (interest group), and not interested in possession in the future (non-interest group) using a self-administered questionnaire. The level of SW acceptance was operationally defined as follows: low (non-interest group), medium (interest group), and high (possessor group). Further, we evaluated the association of acceptance and purchase intentions of SWs with sociodemographic variables, technology literacy, and health variables. Among the participants, 4.2% possessed SWs, with no significant sex difference (men, 4.2%; women, 4.3%). Among men, age < 75 years, obesity, diabetes, and dyslipidemia were significantly associated with SW acceptance level. Contrastingly, among women, age < 75 years, living alone, higher household income, and a high score for new device use in the technology literacy category were significantly associated with SW acceptance level. Health-related factors were associated with SW acceptance in men, while technology literacy and sociodemographic factors were associated with SW acceptance in women. Our findings may inform the development of sex-specific interventions and policies for increasing SW utilization among older adults in Japan.

## 1 Introduction

Smartwatches (SW) are widely recognized as wearable devices that support daily life. According to the Allied Market Research (1), the market size of SW was estimated at $206.4 billion in 2019 and is projected to reach $963.1 billion by 2027. SW are small, autonomous, and non-invasive devices that typically house an accelerometer; they can provide physiological indicators (2). Real-time health information tracking by SW provides useful information that can prompt adopting appropriate daily activities and behaviors. These benefits may help prevent diseases and promote health among older adults (3–8).

Although >60% of older adults are interested in SW, only a small proportion are actual users (9). For nonusers, the barriers to SW use can be an obstacle to promoting self-management of health (10). Therefore, elucidating factors related to SW acceptance and purchase intentions among older adults may help promote effective health management using digital health technologies.

Acceptance of SW is influenced by sex (11), race and cultural background (12). Therefore, to increase the usage of SW, sex- and race-specific analyses are needed. Moreover, sex differences in acceptance and purchase intentions of SW among older Japanese individuals remain unclear.

Therefore, we aimed to investigate the factors associated with the acceptance and purchase intentions of SWs among older adults in Japan. We also examined how those factors vary by sex.

## 2 Methods

### 2.1 Study design and participants

This cross-sectional study used data from the 2022 Itabashi Longitudinal Study on Aging, an ongoing cohort study conducted by the Tokyo Metropolitan Institute for Geriatrics and Gerontology. Itabashi is one of 23 special wards of Tokyo, Japan, with an area of 32.22 km^2^, a total population of 583,608 (population density 18,113/km^2^, as of 1 April 2023) and is formed from 134 districts. Within the Itabashi ward, Takashimadaira is a separate area. Due to urban planning and other factors, it has a higher aging population percentage and single older adult households than the other parts of Itabashi ward (13). The Itabashi Longitudinal Study on Aging is a comprehensive survey of individuals aged ≥65 years living in 37 districts of the Itabashi ward, including the Takashimadaira area.

**Figure 1:**
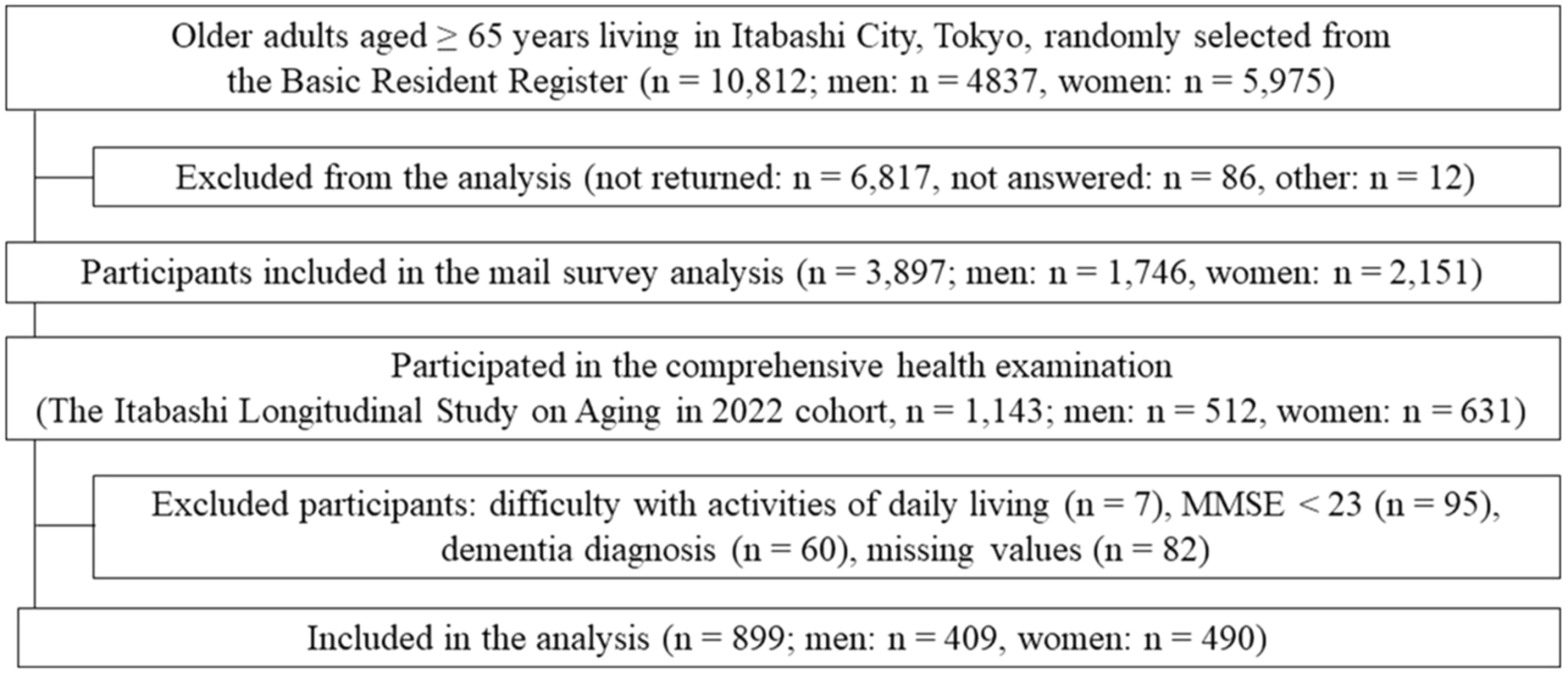
Study flowchart.

A random mail survey was conducted among 10,812 residents in the designated areas, resulting in 3,897 respondents who were subsequently invited to participate in an on-site field survey. From this group, 1,143 individuals participated in the field survey. Among the participants, 162 individuals were excluded due to difficulties in activities of daily living (*n* = 7), a Mini-Mental State Examination (MMSE) score < 23 (*n* = 95), and a dementia diagnosis (*n* = 60). Additionally, 82 individuals were excluded due to missing data. Ultimately, 899 individuals were included (Figure 1).

### 2.2 Possession of SW and purchase intentions among non-possessors

**Figure 2:**
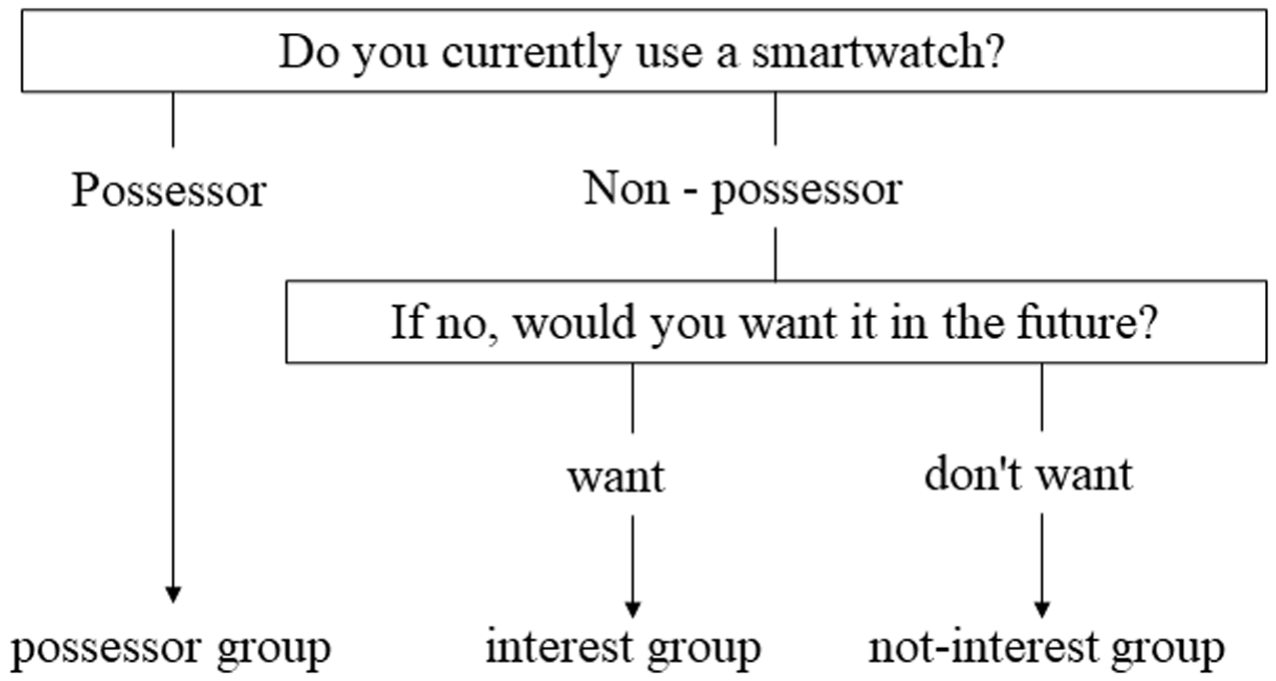
Flow of smartwatch acceptance and willingness to purchase.

We assessed the possession of SW and purchase intentions among non-possessors. Specifically, the following questions were asked: “Do you have a smartwatch? If not, would you like to have one in the future?” Responses were categorized into three groups: “I have one” (possessor group), “I do not have one but would like to have one in the future” (interest group), and “I do not have one and do not want to have either in the future” (non-interest group) (Figure 2). The level of SW acceptance was operationally defined as follows: low (non-interest group), medium (interest group), and high (possessor group). Further, purchase intentions were categorized as low (non-interest group), and high (interest group).

### 2.3 Factors related to the level of acceptance and purchase intentions in SW

Based on a previous research model for wearable device use among older adults (14), we examined categories related to interest in SW, including socio-demographics, technology literacy, and health variables. The sociodemographic characteristics included age, education, employment status, household income, and cohabitation status. Technology literacy refers to the ability to use new devices to obtain information for life management. The technology literacy section of the survey also comprised social participation and interaction opportunities that influence the acceptance of wearable devices (15). Health variables were assessed based on lifestyle (physical activity, dietary diversity, and sleep quality), obesity and chronic diseases (high blood pressure, diabetes, and dyslipidemia), general health status (subjective health status, mild cognitive decline, and depressive symptoms), and the walking speed test.

### 2.4 Socio-demographics

Age was dichotomized using a threshold of 75 years. Education was categorized into high school or lower, junior college or vocational school, college or higher, and other options, with high school or lower being the threshold. Employment status was categorized into full-time (working ≥35 h per week), part-time (working <35 h per week), and unemployed, with employed or unemployed being the thresholds. Household income (JPY) was categorized as follows: no income; < 1 million; 1–3 million; 3–7 million; 7–10 million; and ≥ 10 million. Previous studies reported that the top 9.7% of older households have household incomes above $75 K or more and that SW ownership is higher in this group (14). In this study, we used JPY 7 million (Approximately $50 K at the current exchange rate), the upper tier of household income similar to previous studies, as the reference value and divided it into two values: ≥ 7 million and < 7 million yen (16). Cohabitation status was dichotomized as living alone or not.

### 2.5 Technology literacy

Wel used the Japan Science and Technology Agency’s Index of Competence (JST–IC), which assesses the ability of older adults to live independently or engage in proactive activities (17, 18). Technology literacy was assessed in terms of the following areas: technology usage (ability to use technology equipment), information practice (ability to gather information on health, literacy, and public interest), life management (ability to manage one’s own and family’s life and expenses), and social engagement (represents the level of interest in community or volunteer activities). The JST–IC score ranges from 0–18, with a higher score indicating more engagement. The participants were asked about socializing opportunities: “How often do you meet or go out with friends or neighbors?” Responses were categorized as less than once a month or never and once a month or more.

### 2.6 Health variables

Physical activity was determined by responses to questions about the frequency of participation in (1) walking/light exercise and (2) regular exercise/sports during the week: daily, at least 5–6 days/week, at least 2–4 days/week, at least once/less than once/week, or no exercise at all. The criterion for physical inactivity was both answers were ‘less than once/week’ for (1) walking/light exercise and (2) regular exercise/sports, otherwise ‘physically active’ (19).

Dietary diversity was assessed using the Dietary Variety Score (20) based on 10 food items: meat, fish/shellfish, eggs, milk, soybean products, green/yellow vegetables, potatoes, fruit, seaweed, and fats/oils. One point was assigned in case of an affirmative response to eating a food item “almost every day”; otherwise, zero points were assigned. The total score ranged from 0 to 10, with scores ≥7 considered high (21).

Sleep quality was measured using the Japanese version of the 18-item Pittsburgh Sleep Quality Index (22, 23), with a score of ≥6 indicating poor sleep quality.

Obesity was indicated by a body mass index ≥25 based on the Japanese guidelines (24). The history of chronic diseases (high blood pressure, diabetes, and dyslipidemia) was assessed through interviews conducted by experienced nurses.

Subjective health status was assessed using the following question: “Generally, which of the following phrases best describes your health?” Responses were categorized as “very healthy,” “healthy,” “fair,” “unhealthy,” and “very unhealthy,” with “very healthy” and “healthy” indicating good health.

Cognitive decline was assessed based on the MMSE (25), with a scores <27 indicating a mild cognitive decline.

Depressive symptoms were assessed using the 15-item Japanese version of the Geriatric Depression Scale (26, 27), with scores ≥5 indicating the presence of depressive symptoms.

The 5-m walking test was performed at a normal walking speed in an 11-m walking course, which comprised a 5-m measurement section sandwiched by two 3-m preliminary courses (28). The walking time (in seconds) in the 5-m measurement section was measured using a stopwatch. Walking speed was calculated by dividing the distance in the 5-m section by the walking time, with a threshold walking speed of 1.0 m/s.

### 2.7 Statistical analysis

We described participant characteristics according to the possessor group, interest group, and non-interest group. A chi-square test was used to analyze sex differences in the possessors (possessor group and non-possessors: a total of interest and non-interest group) and willingness to purchase (interest and non-interest group). To clarify factors related to level of acceptance in SW, we conducted ordinal logistic regression analyses, with to level of acceptance in SW as the dependent variable and demographics, technology literacy, and health variables as the explanatory variables. We adjusted for the region (“Takashimadaira area” and “outside Takashimadaira area”) as a confounding variable. Additionally, we conducted binomial logistic regression analyses for interest and non-interest groups to examine intentions for purchasing SW among non-possessors. All results are presented as the adjusted odds ratio and 95% confidence intervals. Statistical significance was set at *p* < 0.05. Statistical analyses were performed using SPSS version 28.0 (IBM Corp., Armonk, NY, United States).

## 3 Results

### 3.1 Characteristics

**Table 1:** Characteristics of the participants according to interest in SW.

	Total	Possessors	Non-Possessors (*n* = 861)	
			Interest	Non-interest
	(*n* = 899)	(*n* = 38)	(*n* = 193)	(*n* = 668)
**Socio-demographics**				
Sex				
Men	409 (45.5)	17 (4.2)	75 (18.3)	317 (77.5)
Women	490 (54.5)	21 (4.3)	118(24.1)	351 (71.6)
Age, < 75 years	283 (31.5)	18 (47.4)	76 (39.4)	189 (28.3)
**Education, graduation**				
Less than high school	799 (88.9)	37 (97.4)	172 (89.1)	590 (88.3)
At least junior college/ college	100 (11.1)	1 (2.6)	21 (10.9)	78 (11.7)
Employment status, employed	254 (28.3)	16 (42.1)	59 (30.6)	179 (26.8)
Household income (JPY), ≥7 mill	73 (8.1)	7 (18.4)	10 (5.2)	56 (8.4)
Living alone	325 (36.2)	12 (31.6)	84 (43.5)	229 (34.3)
**Technology literacy**				
Intellectual tasks (JST-IC), score	3.5 (0.9)	3.8 (0.4)	3.6 (0.8)	3.4 (1.0)
Intellectual curiosity (JST-IC), score	3.5 (0.9)	3.5 (0.9)	3.7 (0.7)	3.4 (0.9)
Information gathering (JST-IC), score	2.9 (1.1)	2.8 (1.1)	3.0 (1.0)	2.9 (1.1)
Creativity (JST-IC), score	1.3 (1.4)	1.3 (1.4)	1.4 (1.4)	1.2 (1.4)
Opportunity to socialize (≥1 wks)	552 (58.1)	28 (73.7)	112 (58.0)	382 (57.2)
**Health variables**				
Physical activity (≥1 wks)	716 (79.8)	31 (81.6)	155 (80.3)	530 (79.3)
Dietary variety (< 5 pts)	362 (40.3)	14 (36.8)	83 (43.0)	265 (39.7)
Sleep quality (PSQI-J score ≥ 6 pts)	595 (66.2)	26 (68.4)	133 (68.9)	436 (65.3)
Obesity (body mass index ≥25 kg/m^2^)	228 (25.4)	13 (34.2)	55 (28.5)	160 (24.0)
High blood pressure, yes	474 (52.7)	21 (55.3)	90 (46.6)	363(54.3)
Diabetes, yes	121 (13.5)	3 (7.9)	36 (18.7)	82 (12.3)
Dyslipidemia, yes	207 (23.0)	5 (13.2)	49 (25.4)	153 (22.9)
Subjective health, healthy	437 (48.6)	22 (57.9)	96 (49.7)	319 (47.8)
Mild cognitive decline (MMSE <27)	338 (37.6)	10 (26.3)	69 (35.8)	259 (38.8)
Depression (GDS-15 ≥ 5 pts)	292 (32.5)	13 (34.2)	61 (31.6)	218 (32.6)
5-m walking speed test, <1.0 m/s	53 (5.9)	2 (5.3)	5 (2.6)	46 (6.9)

Data are presented as mean (SD) or *n* (%). JST-IC, Japan Science and Technology Agency Index of Competence; GDS, Japanese version of the Geriatric Depression Scale; PSQI-J, Japanese version of the Pittsburgh Sleep Quality Index.

The mean age of the included older adults was 77.7 ± 5.0 years; 54.5% of the participants were women. There were 38 (4.2%) SW possessors (Table 1). Among 861 (95.8%) non-possessors, 193 (21.5%) participants were in the interest groups. Additionally, the interest group had a higher proportion of women (25.2%) than men (19.1%) (*p* = 0.035). However, there was no significant between-sex difference in the proportion of SW possessors (men, 4.2%; women, 4.3%; *p* = 0.924).

### 3.2 Level of acceptance in SW

Table 2 summarizes the factors related to SW acceptance. Overall, the level of acceptance of SW was significantly associated with younger age (< 75 years), diabetes, and high scores for new device use and gathering information. Among men, younger age, obesity, diabetes, and dyslipidemia were significantly associated with SW acceptance. Contrastingly, among women, younger age, living alone, a high score for new device use was significantly associated with SW acceptance. Household income of <7 million yen for men and ≥ 7 million yen for women were associated with higher levels of SW acceptance.

**Table 2:** Sex differences in factors related to level of acceptance in smartwatches.

	Total		Men		Women	
	Crude	Adjusted*	Crude	Adjusted*	Crude	Adjusted*
	OR (95% CI)	OR (95% CI)	OR (95% CI)	OR (95% CI)	OR (95% CI)	OR (95% CI)
**Socio-demographics**						
Age, < 75 years (vs. ≥ 75 years)	1.78 (1.31, 2.43)	1.76 (1.25, 2.49)	1.91 (1.19, 3.07)	2.11 (1.21, 3.69)	1.73 (1.15, 2.61)	1.69 (1.05, 2.71)
Education, ≥ (junior) college graduate, (vs. no)	1.30 (0.79, 2.14)	1.01 (0.58, 1.74)	1.39 (0.64, 3.00)	1.06 (0.44, 2.55)	1.31 (0.66, 2.59)	0.94 (0.45, 1.97)
Employment status, yes (vs. no)	1.37 (0.99, 1.89)	1.13 (0.79, 1.62)	1.68 (1.05, 2.69)	1.43 (0.84, 2.45)	1.27 (0.80, 2.00)	0.92 (0.55, 1.56)
Household income (JPY), ≥ 7 mill (vs. < 7 mill)	0.95 (0.55, 1.65)	0.80 (0.44, 1.45)	0.44 (0.17, 1.12)	0.22 (0.08, 0.61)	2.09 (0.99, 4.38)	2.54 (1.13, 5.71)
Cohabitation status, alone (vs. others)	1.31 (0.96, 1.77)	1.31 (0.93, 1.86)	0.97 (0.56, 1.67)	0.70 (0.37, 1.33)	1.41 (0.95, 2.08)	1.98 (1.23, 3.18)
**Technology literacy**						
Intellectual tasks (JST-IC), score	1.51 (1.23, 1.86)	1.36 (1.09, 1.70)	1.43 (1.01, 2.03)	1.28 (0.86, 1.89)	1.62 (1.25, 2.09)	1.50 (1.13, 1.98)
Intellectual curiosity (JST-IC), score	1.44 (1.17, 1.76)	1.31 (1.05, 1.64)	1.47 (1.07, 2.02)	1.40 (0.99, 1.99)	1.40 (1.07, 1.83)	1.18 (0.88, 1.59)
Information gathering (JST-IC), score	1.13 (0.98, 1.30)	0.99 (0.83, 1.17)	1.04 (0.84, 1.29)	0.87 (0.67, 1.13)	1.20 (0.99, 1.46)	1.14 (0.89, 1.46)
Creativity (JST-IC), score	1.10 (0.99, 1.21)	1.09 (0.97, 1.23)	1.15 (0.99, 1.34)	1.14 (0.94, 1.39)	1.06 (0.92, 1.22)	1.05 (0.89, 1.24)
Opportunity to socialize (≥1 wks)	1.17 (0.87, 1.59)	1.00 (0.71, 1.40)	1.31 (0.82, 2.08)	1.09 (0.62, 1.91)	0.99 (0.66, 1.49)	0.85 (0.54, 1.36)
**Health variables**						
Physical activity, score	1.06 (0.73, 1.54)	0.94 (0.63, 1.41)	1.26 (0.72, 2.21)	1.09 (0.58, 2.06)	0.88 (0.53, 1.45)	0.68 (0.39, 1.18)
Good dietary variety (vs. bad)	1.09 (0.80, 1.47)	1.02 (0.67, 1.53)	1.02 (0.61, 1.70)	1.25 (0.62, 2.51)	1.02 (0.69, 1.51)	0.84 (0.49, 1.43)
Sleep quality, no (vs. yes)	0.85 (0.62, 1.17)	0.76 (0.55, 1.06)	1.09 (0.67, 1.76)	0.79 (0.47, 1.34)	0.72 (0.47, 1.11)	0.70 (0.44, 1.10)
Obesity (vs. non-obesity)	1.33 (0.95, 1.85)	1.36 (0.96, 1.93)	1.80 (1.12, 2.89)	2.07 (1.24, 3.45)	1.12 (0.68, 1.83)	1.02 (0.59, 1.75)
High blood pressure, yes (vs. no)	0.79 (0.59, 1.06)	1.02 (0.67, 1.55)	0.78 (0.49, 1.24)	1.14 (0.56, 2.32)	0.84 (0.57, 1.24)	1.10 (0.64, 1.91)
Diabetes, yes (vs. no)	1.38 (0.91, 2.08)	1.59 (1.03, 2.44)	1.44 (0.75, 2.75)	2.10 (1.02, 4.30)	1.32 (0.77, 2.26)	1.33 (0.75, 2.35)
Dyslipidemia, yes (vs. no)	0.99 (0.69, 1.40)	0.99 (0.69, 1.44)	1.47 (0.87, 2.50)	1.89 (1.05, 3.40)	0.72 (0.45, 1.16)	0.63 (0.38, 1.05)
Subjective health (vs. unhealthy)	1.16 (0.86, 1.56)	0.99 (0.72, 1.37)	1.46 (0.92, 2.33)	1.30 (0.78, 2.18)	1.002 (0.68, 1.48)	0.84 (0.54, 1.30)
Mild cognitive declines, yes (vs. no)	0.81 (0.59, 1.10)	1.06 (0.71, 1.56)	0.62 (0.38, 1.01)	0.86 (0.47, 1.57)	1.02 (0.68, 1.55)	1.28 (0.75, 2.19)
Depression symptoms, no (vs. yes)	1.03 (0.75, 1.41)	1.09 (0.78, 1.51)	0.91 (0.56, 1.48)	1.14 (0.67, 1.96)	1.04 (0.69, 1.59)	1.05 (0.67, 1.65)
5-m walking speed test, <1.0 m/s (vs. ≥1.0 m/s)	2.31 (1.04, 5.16)	2.04 (0.89, 4.67)	1.61 (0.54, 4.76)	1.92 (0.59, 6.22)	3.24 (0.97, 10.8)	2.52 (0.73, 8.74)

OR, odds ratio; CI, confidence interval; JST-IC, Japan Science and Technology Agency Index of Competence. *Region.

### 3.3 Purchase intention for SW among non-possessors

Table 3 summarizes the factors related to purchase intentions for SW among non-possessors. In the overall population, younger age, diabetes, and high scores for new device use and gathering information were associated with high purchase intentions for SW. Among men, younger age, a household income <7 million yen, obesity, diabetes, and dyslipidemia were associated with high purchase intentions. In contrast, living alone and high scores for new device use and gathering information were associated with high purchase intentions among women.

**Table 3:** Sex differences in the purchase intentions for smartwatches.

	Total		Men		Women	
	Crude	Adjusted*	Crude	Adjusted*	Crude	Adjusted*
	OR (95% CI)	OR (95% CI)	OR (95% CI)	OR (95% CI)	OR (95% CI)	OR (95% CI)
**Socio-demographics**						
Age, < 75 years (vs. ≥ 75 years)	1.68 (1.20, 2.34)	1.78 (1.22, 2.59)	2.01 (1.20, 3.36)	2.57 (1.38, 4.80)	1.52 (0.97, 2.36)	1.52 (0.91, 2.55)
Education, ≥ (junior) college graduate, (vs. no)	1.14 (0.68, 1.92)	0.83 (0.48, 1.46)	1.06 (0.49, 2.28)	0.80 (0.33, 1.94)	1.18 (0.59, 2.40)	0.82 (0.38, 1.79)
Employment status, yes (vs. no)	1.23 (0.87, 1.75)	1.07 (0.72, 1.59)	1.53 (0.92, 2.56)	1.36 (0.75, 2.49)	0.83 (0.51, 1.36)	0.88 (0.50, 1.57)
Household income (JPY), ≥ 7 mill (vs. < 7 mill)	0.60 (0.30, 1.19)	0.51 (0.25, 1.07)	0.20 (0.05, 0.83)	0.09 (0.02, 0.42)	1.42 (0.60, 3.39)	1.78 (0.69, 4.55)
Cohabitation status, alone (vs. others)	1.46 (1.05, 2.01)	1.43 (0.99, 2.09)	1.16 (0.65, 2.05)	0.94 (0.48, 1.84)	1.49 (0.98, 2.26)	1.86 (1.11, 3.10)
**Technology literacy**						
Intellectual tasks (JST-IC), score	1.44 (1.16, 1.78)	1.33 (1.06, 1.68)	1.39 (0.96, 2.02)	1.32 (0.86, 2.02)	1.52 (1.17, 1.97)	1.46 (1.09, 1.94)
Intellectual curiosity (JST-IC), score	1.58 (1.25, 2.00)	1.47 (1.14, 1.90)	1.46 (1.04, 2.05)	1.37 (0.94, 2.01)	1.68 (1.22, 2.32)	1.46 (1.02, 2.09)
Information gathering (JST-IC), score	1.17 (0.998, 1.37)	1.06 (0.88, 1.29)	1.09 (0.86, 1.38)	0.96 (0.72, 1.29)	1.23 (0.995, 1.52)	1.18 (0.90, 1.54)
Creativity (JST-IC), score	1.10 (0.99, 1.23)	1.13 (0.99, 1.28)	1.19 (1.01, 1.40)	1.25 (1.00, 1.55)	1.05 (0.90, 1.23)	1.07 (0.89, 1.28)
Opportunity to socialize (≥1 week)	1.02 (0.74, 1.41)	0.85 (0.59, 1.22)	1.25 (0.75, 2.07)	0.95 (0.50, 1.77)	0.80 (0.52, 1.23)	0.69 (0.42, 1.12)
**Health variables**						
Physical activity, score	1.04 (0.70, 1.55)	0.94 (0.61, 1.46)	1.32 (0.71, 2.46)	1.25 (0.62, 2.52)	0.81 (0.48, 1.37)	0.65 (0.36, 1.16)
Good dietary variety (vs. bad)	1.15 (0.83, 1.58)	1.33 (0.83, 2.14)	1.02 (0.59, 1.77)	1.49 (0.67, 3.32)	1.09 (0.72, 1.66)	1.21 (0.65, 2.25)
Sleep quality, no (vs. yes)	0.85 (0.60, 1.20)	0.77 (0.54, 1.11)	1.11 (0.66, 1.87)	0.84 (0.47, 1.51)	0.71 (0.45, 1.12)	0.71 (0.43, 1.16)
Obesity (vs. non-obesity)	1.25 (0.88, 1.80)	1.31 (0.89, 1.92)	1.76 (1.06, 2.95)	2.04 (1.15, 3.60)	1.04 (0.61, 1.79)	0.98 (0.54, 1.77)
High blood pressure, yes (vs. no)	0.73 (0.53, 1.01)	0.91 (0.57, 1.46)	0.74 (0.45, 1.23)	1.02 (0.45, 2.32)	0.77 (0.51, 1.17)	0.96 (0.53, 1.74)
Diabetes, yes (vs. no)	1.64 (1.07, 2.51)	1.88 (1.20, 2.96)	1.59 (0.80, 3.17)	2.62 (1.19, 5.77)	1.64 (0.94, 2.85)	1.66 (0.92, 2.97)
Dyslipidemia, yes (vs. no)	1.13 (0.78, 1.64)	1.16 (0.78, 1.72)	1.72 (0.98, 3.02)	2.31 (1.22, 4.39)	0.83 (0.51, 1.36)	0.72 (0.42, 1.23)
Subjective health (vs. unhealthy)	1.08 (0.79, 1.49)	0.94 (0.66, 1.33)	1.36 (0.82, 2.25)	1.15 (0.65, 2.04)	0.95 (0.62, 1.44)	0.83 (0.52, 1.34)
Mild cognitive declines, yes (vs. no)	0.88(0.63, 1.22)	1.21 (0.79, 1.85)	0.70 (0.42, 1.18)	0.97 (0.50, 1.89)	1.09 (0.71, 1.69)	1.52 (0.86, 2.69)
Depression, no (vs. yes)	1.06 (0.75, 1.49)	1.16 (0.81, 1.67)	1.23 (0.72, 2.11)	1.32 (0.73, 2.41)	0.93 (0.59, 1.45)	1.09 (0.67, 1.76)
5-m walking speed test, <1.0 m/s (vs. ≥1.0 m/s)	2.78 (1.09, 7.10)	1.57 (0.90, 2.75)	1.78 (0.52, 6.12)	1.77 (0.72, 4.41)	4.27 (0.993, 18.3)	1.73 (0.79, 3.76)

OR, odds ratio; CI, confidence interval; JST-IC, Japan Science and Technology Agency Index of Competence.*Region.

## 4 Discussion

In the overall population, age, intellectual tasks, intellectual curiosity of technology literacy, and diabetes were associated with SW acceptance. Among men, health variables such as obesity, diabetes, and dyslipidemia were associated with SW acceptance. Contrastingly, demographic factors such as the ability to use technology, living alone, and household income were associated with high acceptance among women. In both sexes, age < 75 years was related to a high level of SW acceptance. Regarding purchase intentions, a similar trend was observed for men. However, among women, age or household income were not related to purchase intentions; instead, a high score for information-gathering skills was a factor in purchase intentions. Taken together, sex-specific interventions are warranted to increase acceptance and purchase intentions for SW.

### 4.1 The proportion of SW possessors among older adult individuals in Japan

Previous research suggests that the proportion of SW ownership is significantly lower among older adults than among younger individuals (29). Surveys exclusively on older populations in the United States and Canada found ownership proportions of 17.5% (14) and 12.3% (30), respectively. Also, women have a higher usage of wearable devices than men (14). Our study addressed the lack of detailed data on the SW ownership prevalence among the older population in Japan. The proportion of SW possession among older Japanese adults in the present study was 4.2%, with no sex differences, and this number was significantly lower than that reported in previous studies. Furthermore, we found a clear inverse relationship between aging and SW acceptance, with the tendency to accept SW decreasing with age. The widespread use of SW may facilitate older adults in Japan to promote their good health.

### 4.2 Sex differences in factors related to SW acceptance

Older adults classified as overweight are more likely to adopt wearable devices (14). Our study further shows that those with obesity and various chronic diseases had a higher SW acceptance, a trend observed predominantly among men. Women are reportedly more interested in health-related information than men and are more attentive to how the products they purchase affect their health (31).

This increased awareness may explain why obesity and chronic diseases, such as diabetes and dyslipidemia, are more strongly correlated with SW acceptance among men. Older adults with chronic diseases are likely to access health information through smartphone apps and the Internet (32). Therefore, equipping SW with features that provide specific guidelines and goal setting for activities and sleep could make these devices particularly beneficial for men in preventing chronic conditions.

Among women, the ability to use new devices, living alone, and high household income were associated with SW acceptance. Older people often struggle with technology anxiety and resistance to change, which hinders their learning and use of digital technology (33, 34). This issue may be particularly pronounced among older Japanese women. Use of wearable devices requires regular support and feedback from healthcare professionals (35). Therefore, to reduce resistance to digital device, it is necessary to develop simple device to operate and establish a unified support system.

Women tend to use wearable devices more regularly than men to monitor their health-related information (36). Moreover, even those who do not own wearable devices have a notable interest in these devices as viable tools for improving physical and mental health (9). Interestingly, contrary to the findings of this study, the use of wearable devices in Finland was higher among married or cohabiting older people than among their single counterparts (37). Among older Japanese women, increased health-related anxieties due to living alone may increase their interest in self-health management, potentially leading to higher acceptance of SW.

### 4.3 Sex differences in the purchase intentions for SW

SW purchase intention among men in Japan was associated with high scores for social engagement in addition to SW acceptance-related items. Opportunities for social participation and interaction are believed to influence the acceptance of SW (38). Older men may be more dependent on peers and family members regarding SW implementation. Thus, social campaigns about SW with family and friends using opportunities for social participation and interaction may lead to SW adoption.

Among women, the use of new equipment was associated with the ability to gather information, but not with social participation. This finding suggests that women may be more influenced by information about SW from information technology devices (e.g., Internet, smartphones, TV) than by social participation. Therefore, featuring SW on TV or other media in connection with health may increase their willingness to purchase SW. In contrast, household income above JPY 7 million was associated with higher SW acceptance among women but not with purchase intention. Women prefer lower-priced SW than men (39). However, women may not be aware of the price of SW at the stage of purchase intention. In our survey, women were more likely than men to have a high purchase intention, but no significant difference was observed in the percentage of ownership. This may be due to the high price of SW. Therefore, it is desirable to investigate the price range of SW desired by older adult women in Japan.

This study has several limitations. First, we only used data from Japanese participants and only one urban region, limiting the generalizability of the findings. For this reason, it is desirable to conduct surveys on a national scale and in non-urban areas. Second, the participants comprised older people who spontaneously participated in a health screening. This group is likely to be more health-conscious and, as a result, may predominantly comprise healthier older adults. This implies the need for further validation in populations with different characteristics. Third, since this was a cross-sectional study, we could not determine the temporal changes in the interest in wearable devices. Therefore, we would like to conduct a longitudinal study. Fourth, older adults’ acceptance of SW suggests that perceived usefulness, compatibility, and facilitating conditions positively affect to the use of such technologies (15). However, these factors have not been empirically examined. Verification of these factors may mention insights in further acceptance. Finally, purchase intention and usage status were self-reported, which could have led to recall bias.

## 5 Conclusion

We observed sex differences in factors associated with interest in SW among older adults. Health variables such as obesity, diabetes, and dyslipidemia were associated with interest in SW among men. Moreover, technology literacy, such as the ability to possess new devices and socio-demographics, such as household income and cohabitation status, were associated with interest in SW among women. Age was the only common factor. Therefore, sex-specific strategies are warranted to promote the use of wearable devices for health management among older adults.

## Data availability statement

The datasets generated and/or analyzed during the current study are available from the corresponding author upon reasonable request.

## Ethics statement

The studies involving humans were approved by the Ethics Committee of the Tokyo Metropolitan Institute for Geriatrics and Gerontology approved this study (approval number: R21-056). The studies were conducted in accordance with the local legislation and institutional requirements. Written informed consent for participation in this study was provided by the participants’ legal guardians/next of kin.

## Author contributions

ND: Formal analysis, Methodology, Writing – original draft, Writing – review & editing. YO: Conceptualization, Data curation, Investigation, Methodology, Writing – review & editing. NK: Conceptualization, Data curation, Investigation, Methodology, Writing – review & editing. KM: Data curation, Investigation, Writing – review & editing. MI: Data curation, Investigation, Writing – review & editing. HI: Data curation, Investigation, Writing – review & editing. FM: Data curation, Investigation, Writing – review & editing. TO: Data curation, Investigation, Writing – review & editing. HH: Data curation, Investigation, Writing – review & editing. SA: Investigation, Writing – review & editing. HS: Funding acquisition, Methodology, Project administration, Resources, Writing – review & editing.

## References

[1] Research, A.M., Smartwatch market by product. Extension: standalone and classical [application] (personal assistance, wellness, healthcare, sports, and others), and operating system (WatchOS, android, RTOS, Tizen, and others): global opportunity analysis and industry forecast (2020–2027).

[2] Wearable medical devices market size, S. a. I. A. b (2019–2026). Fortune business insights. P.D.P.M., therapeutics, by application (remote patient monitoring and home healthcare, sports and fitness), by distribution channel (retail pharmacies, online pharmacies, hypermarkets) and regional forecast. Available at: https://www.fortunebusinessinsights.com/industry-reports/wearable-medical-devices-market-101070 (Accessed Aug 08, 2020) (2019).

[3] Quiroz JC Geangu E Yong MH. Emotion recognition using smart watch sensor data: mixed-design study. JMIR Ment Health. (2018) 5:e10153. doi: 10.2196/10153, PMID: 30089610 PMC6105867

[4] Straiton N Alharbi M Bauman A Neubeck L Gullick J Bhindi R. The validity and reliability of consumer-grade activity trackers in older, community-dwelling adults: a systematic review. Maturitas. (2018) 112:85–93. doi: 10.1016/j.maturitas.2018.03.016, PMID: 29704922

[5] Kononova A Li L Kamp K Bowen M Rikard RV Cotten S. The use of wearable activity trackers among older adults: focus group study of tracker perceptions, motivators, and barriers in the maintenance stage of behavior change. JMIR Mhealth Uhealth. (2019) 7:e9832. doi: 10.2196/mhealth.9832, PMID: 30950807 PMC6473213

[6] Jo A Coronel BD Coakes CE Mainous AG 3rd. Is there a benefit to patients using wearable devices such as Fitbit or health apps on mobiles? A systematic review. Am J Med. (2019) 132:1394–1400.e1. doi: 10.1016/j.amjmed.2019.06.018, PMID: 31302077

[7] Stavropoulos TG Papastergiou A Mpaltadoros L Nikolopoulos S Kompatsiaris I. IoT wearable sensors and devices in elderly care: a literature review. Sensors (Basel). (2020) 20:2826. doi: 10.3390/s20102826, PMID: 32429331 PMC7288187

[8] Master H Annis J Huang S Beckman JA Ratsimbazafy F Marginean K. Association of step counts over time with the risk of chronic disease in the all of US Research program. Nat Med. (2022) 28:2301–8. doi: 10.1038/s41591-022-02012-w, PMID: 36216933 PMC9671804

[9] Kekade S Hseieh CH Islam MM Atique S Mohammed Khalfan A Li YC. The usefulness and actual use of wearable devices among the elderly population. Comput Methods Prog Biomed. (2018) 153:137–59. doi: 10.1016/j.cmpb.2017.10.008, PMID: 29157447

[10] Kang HS Exworthy M. Wearing the future-wearables to empower users to take greater responsibility for their health and care: scoping review. JMIR Mhealth Uhealth. (2022) 10:e35684. doi: 10.2196/35684, PMID: 35830222 PMC9330198

[11] Zhang M Luo M Nie R Zhang Y. Technical attributes, health attribute, consumer attributes and their roles in adoption intention of healthcare wearable technology. Int J Med Inform. (2017) 108:97–109. doi: 10.1016/j.ijmedinf.2017.09.016, PMID: 29132639

[12] Yang Meier D Barthelmess P Sun W Liberatore F. Wearable technology acceptance in health care based on national culture differences: cross-country analysis between Chinese and Swiss consumers. J Med Internet Res. (2020) 22:e18801. doi: 10.2196/18801, PMID: 33090108 PMC7644382

[13] Itabashi-ku home page. Takashimadaira area grand design has been formulated. Takashimadaira Area Grand Design has been formulated|Itabashi Ward Official Website. Available at: https://j-server.com.

[14] Chandrasekaran R Katthula V Moustakas E. Too old for technology? Use of wearable healthcare devices by older adults and their willingness to share health data with providers. Health Informatics J. (2021) 27:146045822110580. doi: 10.1177/14604582211058073 34802315

[15] Li J Ma Q Chan AH Man SS. Health monitoring through wearable technologies for older adults: smart wearables acceptance model. Appl Ergon. (2019) 75:162–9. doi: 10.1016/j.apergo.2018.10.006, PMID: 30509522

[16] Ministry of Internal Affairs and Communications: family income and expenditure survey. Available at: http://www.stat.go.jp/english/data/kakei/index.htm.

[17] Iwasa H Masui Y Inagaki H Yoshida Y Shimada H Otsuka R. Development of the Japan Science and Technology Agency index of competence to assess functional capacity in older adults: conceptual definitions and preliminary items. Gerontol Geriatr Med. (2015) 1:233372141560949. doi: 10.1177/2333721415609490 PMC5119882 28138472

[18] Iwasa H Masui Y Inagaki H Yoshida Y Shimada H Otsuka R. Assessing competence at a higher level among older adults: development of the Japan Science and Technology Agency index of competence (JST-IC). Aging Clin Exp Res. (2018) 30:383–93. doi: 10.1007/s40520-017-0786-8, PMID: 28646250

[19] Satake S Arai H. The. Rev. Japanese version of the cardiovascular health study criteria (revised J-CHS criteria). Geriatr Gerontol Int. (2020) 20:992–3. doi: 10.1111/ggi.14005, PMID: 33003255

[20] Kumagai S Watanabe S Shibata H Amano H Fujiwara Y Shinkai S. Effects of dietary variety on declines in high-level functional capacity in elderly people living in a community. Nihon Koshu Eisei Zasshi. (2003) 50:1117–24. PMID: 14750363

[21] YokoyamaYNishiMMurayamaHAmanoHTaniguchiYNofujiY. Dietary variety and decline in lean mass and physical performance in community-dwelling older Japanese: a 4-year follow-up study. J Nutr Health Aging. (2017) 21:11–6. doi: 10.1007/s12603-016-0726-x, PMID: 27999844

[22] Smyth CA. Evaluating sleep quality in older adults: the Pittsburgh sleep quality index can be used to detect sleep disturbances or deficits. Am J Nurs. (2008) 108:42–50. doi: 10.1097/01.NAJ.0000317300.33599.63 18434798

[23] Doi Y Minowa M Uchiyama M Okawa M Kim K Shibui K. Psychometric assessment of subjective sleep quality using the Japanese version of the Pittsburgh sleep quality index (PSQI-J) in psychiatric disordered and control subjects. Psychiatry Res. (2000) 97:165–72. doi: 10.1016/S0165-1781(00)00232-8, PMID: 11166088

[24] Japan Society for the Study of Obesity. Novel criteria for “obesity disease” in Japan on the recommendation of Japan society for the study of obesity. J Jpn Soc Stud Obes. (2000) 6:18–28.

[25] Chun CT Seward K Patterson A Melton A MacDonald-Wicks L. Evaluation of available cognitive tools used to measure mild cognitive decline: a scoping review. Nutrients. (2021) 13:3974. doi: 10.3390/nu13113974, PMID: 34836228 PMC8623828

[26] Almeida OP Almeida SA. Short versions of the geriatric depression scale: a study of their validity for the diagnosis of a major depressive episode according to ICD-10 and DSM-IV. Int J Geriatr Psychiatry. (1999) 14:858–65. doi: 10.1002/(SICI)1099-1166(199910)14:10<858::AID-GPS35>3.0.CO;2-8, PMID: 10521885

[27] Sugishita K Sugishita M Hemmi I Asada T Tanigawa T. A validity and reliability study of the Japanese version of the geriatric depression scale 15 (GDS-15-J). Clin Gerontol. (2017) 40:233–40. doi: 10.1080/07317115.2016.1199452, PMID: 28452641

[28] Clegg A Young J Iliffe S Rikkert MO Rockwood K. Frailty in elderly people. Lancet. (2013) 381:752–62. doi: 10.1016/S0140-6736(12)62167-9, PMID: 23395245 PMC4098658

[29] Chandrasekaran R Katthula V Moustakas E. Patterns of use and key predictors for the use of wearable health care devices by US adults: insights from a National Survey. J Med Internet Res. (2020) 22:e22443. doi: 10.2196/22443, PMID: 33064083 PMC7600024

[30] Jaana M Paré G. Comparison of mobile health technology use for self-tracking between older adults and the general adult population in Canada: cross-sectional survey. JMIR Mhealth Uhealth. (2020) 8:e24718. doi: 10.2196/24718, PMID: 33104517 PMC7717921

[31] Ek S. Gender differences in health information behaviour: a Finnish population-based survey. Health Promot Int. (2015) 30:736–45. doi: 10.1093/heapro/dat063, PMID: 23985248

[32] Asan O Cooper F Nagavally S Walker RJ Williams JS Ozieh MN. Preferences for health information technologies among US adults: analysis of the health information national trends survey. J Med Internet Res. (2018) 20:31. doi: 10.2196/jmir.9436 PMC6245956 30341048

[33] Talukder MS Sorwar G Bao Y Ahmed JU Palash MAS. Predicting antecedents of wearable healthcare technology acceptance by elderly: a combined SEM-neural network approach. Technol Forecast Soc Change. (2020) 150:119793. doi: 10.1016/j.techfore.2019.119793

[34] Hoque R Sorwar G. Understanding factors influencing the adoption of mHealth by the elderly: an extension of the UTAUT model. Int J Med Inform. (2017) 101:75–84. doi: 10.1016/j.ijmedinf.2017.02.002, PMID: 28347450

[35] Brickwood KJ Williams AD Watson G O'Brien J. Older adults' experiences of using a wearable activity tracker with health professional feedback over a 12-month randomised controlled trial. Digit Health. (2020) 6:205520762092167. doi: 10.1177/2055207620921678 PMC7218318 32426152

[36] Del Busso L Brottveit G Torp Løkkeberg S Gluppe G. Women’s embodied experiences of using wearable digital self-tracking health technology: a review of the qualitative research literature. Health Care Women Int. (2022) 43:1355–79. doi: 10.1080/07399332.2021.1884682, PMID: 33900152

[37] Kyytsönen M Vehko T Anttila H Ikonen J. Factors associated with use of wearable technology to support activity, well-being, or a healthy lifestyle in the adult population and among older adults. PLOS Digit Health. (2023) 2:e0000245. doi: 10.1371/journal.pdig.0000245, PMID: 37163490 PMC10171588

[38] Lazaro MJS Lim J Kim SH Yun MH. Wearable technologies: acceptance model for smartwatch adoption among older adults. International Conference on Human-Computer Interaction. (2020) p. 303–315. Available at: https://link.springer.com/chapter/10.1007/978-3-030-50252-2_23.

[39] Gupta M Sinha N Singh P Chuah SHW. Gender differences in the wearable preferences, device and advertising value perceptions: smartwatches vs. fitness trackers. Int J Technol Mark. (2020) 14:199–225. doi: 10.1504/IJTMKT.2020.110127

