# Corrigendum: Sex-specific factors associated with acceptance of smartwatches among urban older adults: the Itabashi longitudinal study on aging

**DOI:** 10.3389/fpubh.2024.1416463

**Published:** 2024-05-15

**Authors:** Naoki Deguchi, Yosuke Osuka, Narumi Kojima, Keiko Motokawa, Masanori Iwasaki, Hiroki Inagaki, Fumiko Miyamae, Tsuyoshi Okamura, Hirohiko Hirano, Shuichi Awata, Hiroyuki Sasai

**Affiliations:** ^1^Research Team for Promoting Independence and Mental Health, Tokyo Metropolitan Institute for Geriatrics and Gerontology, Tokyo, Japan; ^2^Department of Frailty Research, Center for Gerontology and Social Science, Research Institute, National Center for Geriatrics and Gerontology, Obu, Japan; ^3^Division of Preventive Dentistry, Department of Oral Health Science, Graduate School of Dental Medicine, Hokkaido University, Sapporo, Japan; ^4^Integrated Research Initiative for Living Well with Dementia, Tokyo Metropolitan Institute for Geriatrics and Gerontology, Tokyo, Japan

**Keywords:** wearable healthcare device, mobile health, smart wearables, health promotion, technology innovativeness

In the published article, there was an error in Figure 1 as published. Some numbers were incorrect in Figure 1. The corrected Figure 1 and its caption appear below.

**Figure 1:**
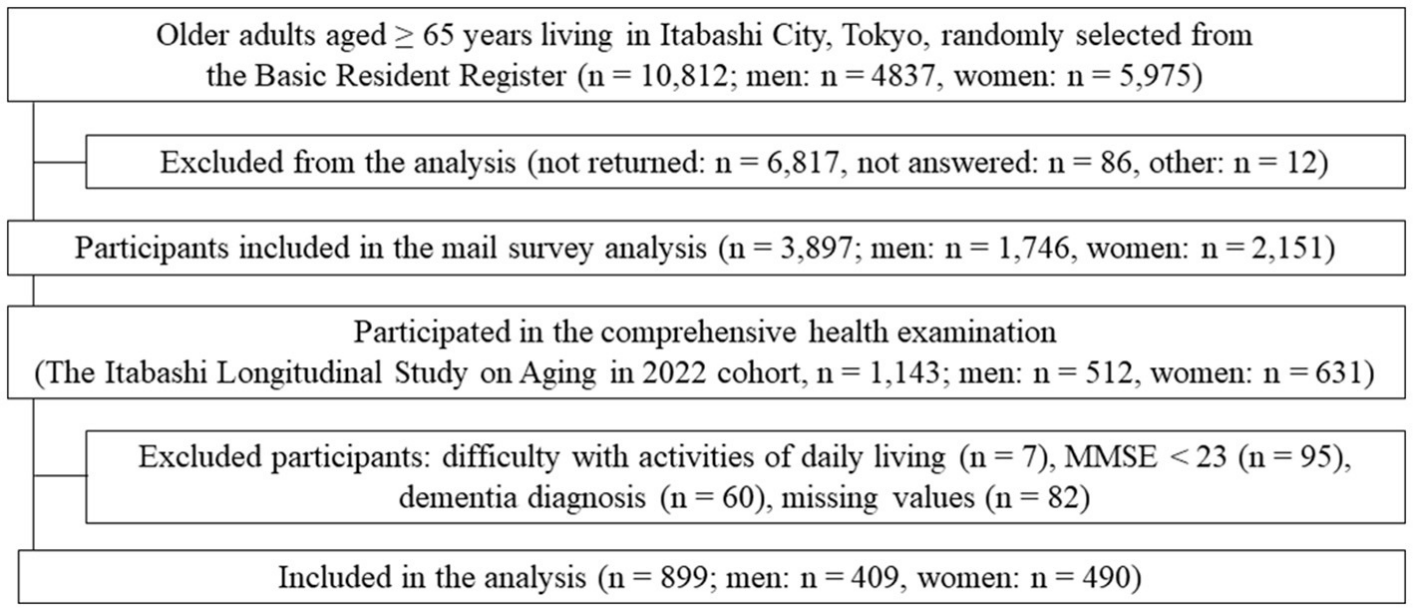
Study flowchart.

The authors apologize for this error and state that this does not change the scientific conclusions of the article in any way. The original article has been updated.

